# Post-traumatic stress disorder and post COVID 19 syndrome

**DOI:** 10.1192/j.eurpsy.2023.498

**Published:** 2023-07-19

**Authors:** N. Halouani, D. Gdoura, N. Bouattour, M. Turki, N. Moussa, S. Ellouze, J. Aloulou

**Affiliations:** ^1^Psychiatry “B” Departement; ^2^Pulmonology Departement, Hedi Chaker Hospital, Sfax, Tunisia

## Abstract

**Introduction:**

Patients hospitalized in the case of COVID19 have had to face a complex and potentially very stressful situation. In this context a screening program for psychological distress in patients with COVID19 is necessary.

**Objectives:**

To screen for post-traumatic stress disorder post COVID19 and to identify the epidemiological and clinical factors correlated with this disorder in post COVID19 patients.

**Methods:**

A descriptive and analytical cross-sectional study that took place during the period from 1^st^ March to 15^th^ May 2021 with 154 patients who were hospitalized at the COVID19 unit at Hedi Chaker Hospital Sfax.

We used a pre-established form to record sociodemographic, clinical and therapeutic data. The post-traumatic stress disorder was assessed by the “Impact of Event Scale-Revised”.

**Results:**

The mean age was 66.62 ± 13.34 years with a male predominance of 60.4%. In our sample, 77.9% of the patients had a somatic history, of which hypertension was the most frequent pathology (46.1%). The average length of hospitalization was 9.5 days ± 6.3. The form was considered severe in 27.9 cases.

According to the IES-R scale, twenty-one patients (13.6%) had post-traumatic stress disorder, with a predominance of women (57.1%).

A significant association was found between marital status and post-traumatic stress disorder. Thus, married or widowed patients are more likely to develop PTSD.

In the present study, we did not find statistically significant associations between the clinical characteristics of the disease (severity of the disease, length of hospitalization, functional signs) and post-traumatic stress disorder.

**Conclusions:**

Psychological support interventions in surviving patients of COVID 19 is necessary aiming to increase resilience, manage coping strategies and decrease the deleterious impact of the pandemic on mental health.

**Disclosure of Interest:**

None Declared

